# Supplementary material for: Lipopolysaccharide and Wzi retain capsular polysaccharide at the Klebsiella pneumoniae cell surface and promote bloodstream infection fitness
Source: bioRxiv. 2026 Oct 31:2026.10.30.762281. Preprint. [Version 1] doi: 10.64898/2026.10.30.762281 (PMC13734125; doi:10.64898/2026.10.30.762281)
Supplement: Supplementary file 1 [file NIHPP2026.10.30.762281v1-supplement-3.pdf]

bioRxiv preprint doi: https://doi.org/10.64898/2026.10.30.762281; this version posted October 31, 2026. The copyright holder for this preprint (which was not certified by peer review) is the author/funder, who has granted bioRxiv a license to display the preprint in perpetuity. It is made available under a CC-BY 4.0 International license.

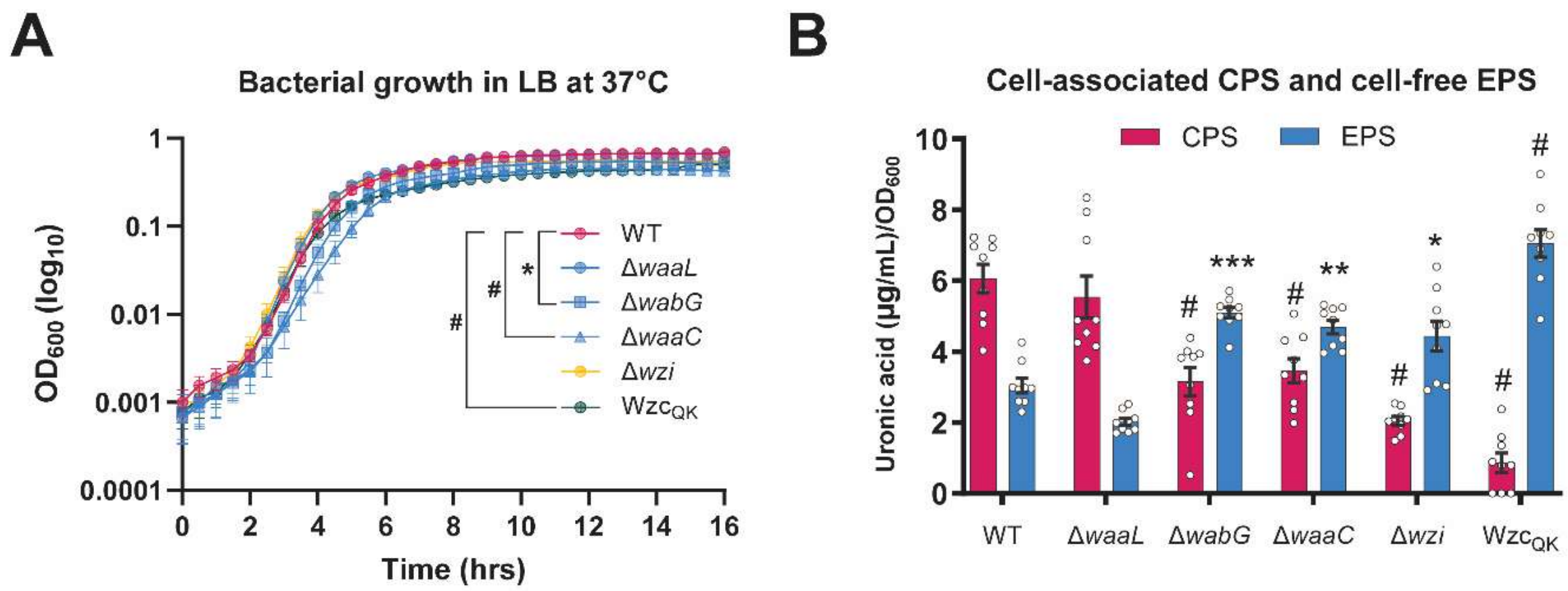

S1 Fig: Deletion of CPS retention components increases proportion of cell-free EPS without impairing *K. pneumoniae* growth *in vitro*.

KPPR1 WT and the mutants were cultured in LB and their cell-associated CPS, cell-free EPS abundance and growth kinetics were analyzed. **(A)** Cell-associated CPS and cell-free EPS abundance were determined by uronic acid quantification. Cell-associated CPS was calculated by subtracting cell-free EPS from total CPS abundance. The corresponding 'total CPS abundance' and '% total CPS attached to the cell surface' are presented in Fig 1C and 19D, respectively. **(B)** Bacterial growth curves were generated by measuring OD_{600} of WT and the mutants every 30 minutes cultured aerobically for 16 hours at 37°C. (A,B) Data presented are the mean, and error bars represent the standard error of the mean. Statistical significance was determined by comparing each mutant to WT using (A) two-way ANOVA with Dunnett's post-hoc test or (B) one-way ANOVA with Dunnett's post-hoc test. *P*-values are displayed (A) above the bars or (B) alongside data legend with vertical brackets, for each comparison; * indicates $p \leq 0.05$, *** indicates $p \leq 0.001$, # p indicates ≤ 0.0001. Experiments were performed three independent times, each in biological triplicate (n = 9).

bioRxiv preprint doi: https://doi.org/10.64898/2026.10.30.762281; this version posted October 31, 2026. The copyright holder for this preprint (which was not certified by peer review) is the author/funder, who has granted bioRxiv a license to display the preprint in perpetuity. It is made available under a CC-BY 4.0 International license.

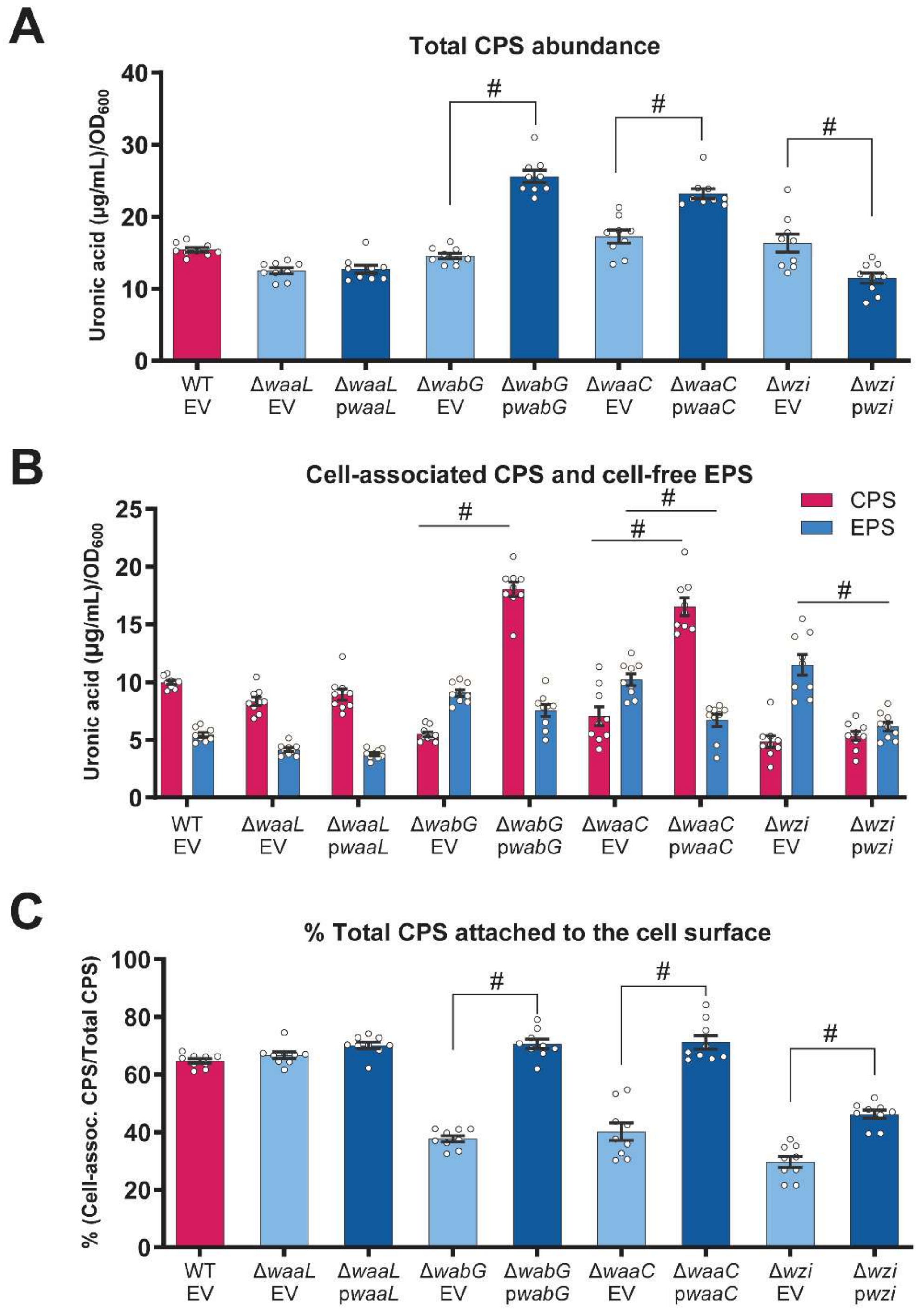

S2 Fig: Genetic complementation of the LPS and *wzi* deletion strains.

KPPR1 mutants Δ*waaL*, Δ*wabG*, Δ*waaC* and Δ*wzi* were genetically complemented using the pBBR1-MCS5 vector carrying the respective endogenous promoter and gene. The strains were cultured in LB and their cell-associated CPS and cell-free EPS abundance were analyzed. **(A)**

bioRxiv preprint doi: https://doi.org/10.64898/2026.10.30.762281; this version posted October 31, 2026. The copyright holder for this preprint (which was not certified by peer review) is the author/funder, who has granted bioRxiv a license to display the preprint in perpetuity. It is made available under a CC-BY 4.0 International license.

Total CPS abundance was determined by measuring uronic acid content in total crude CPS extracts and normalized to OD_{600}. **(B)** Cell-associated CPS and cell-free EPS abundance were determined by uronic acid quantification. Cell-associated CPS was calculated by subtracting cell-free EPS from total CPS abundance. **(C)** The percentage of total CPS retained on the bacterial cell surface was calculated as the ratio of cell-associated CPS to total CPS shown in (B). Data presented are the mean, and error bars represent the standard error of the mean. Statistical significance was determined by comparing adjacent empty vector (EV) and complementation strains to WT using two-way ANOVA with Šídák correction for multiple comparisons. *P*-values are displayed above the bars with connecting lines; * indicates $p \leq 0.05$, ** indicates $p \leq 0.01$, # indicates $p \leq 0.0001$. Experiments were performed three independent times, each in biological triplicate ($n = 9$). 'EV' indicates strain with an empty pBBR1 vector and p*gene* indicates strain with pBBR1 encoding the gene under its respective endogenous promoter.

bioRxiv preprint doi: https://doi.org/10.64898/2026.10.30.762281; this version posted October 31, 2026. The copyright holder for this preprint (which was not certified by peer review) is the author/funder, who has granted bioRxiv a license to display the preprint in perpetuity. It is made available under a CC-BY 4.0 International license.

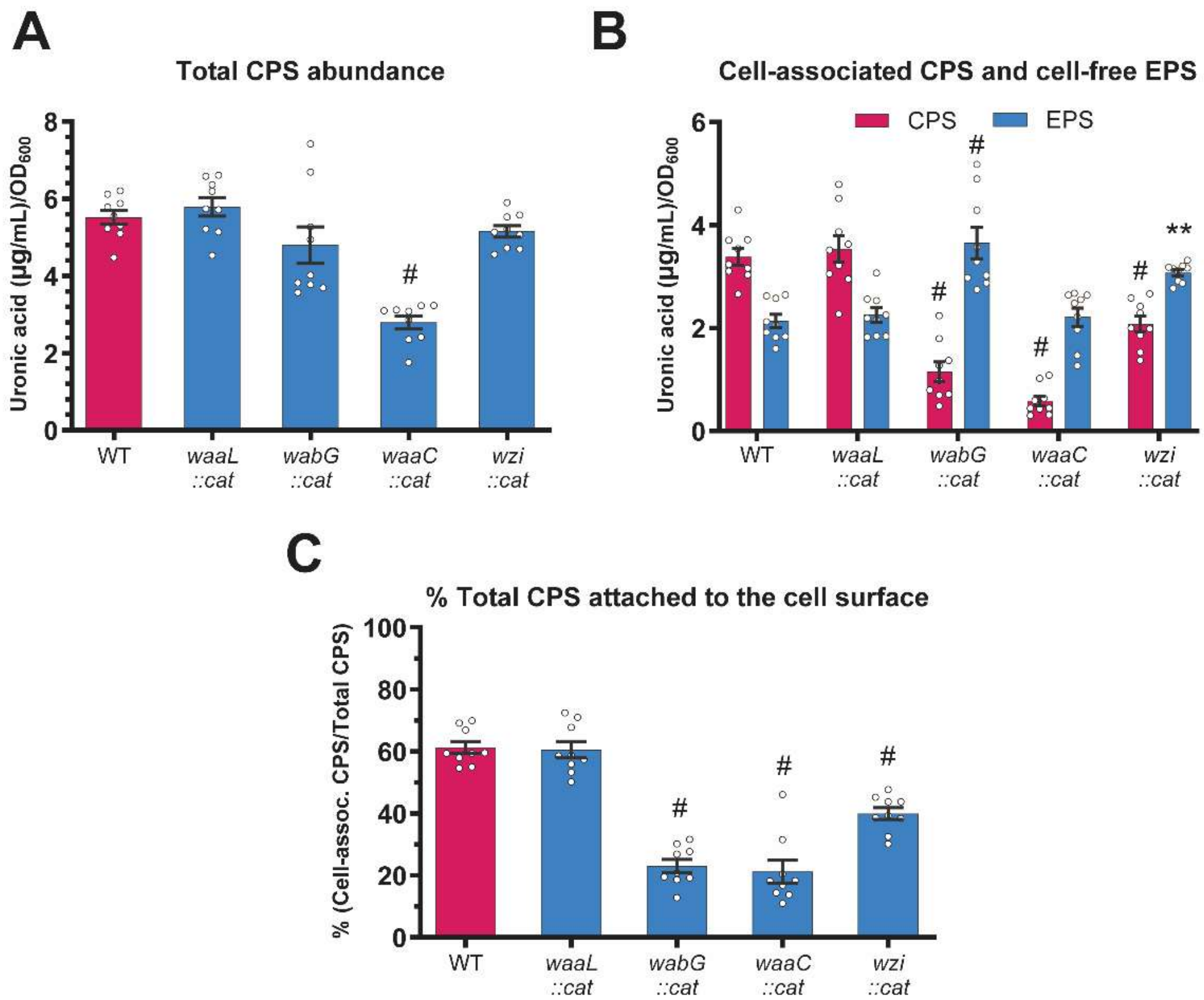

S3 Fig: A classical *K. pneumoniae* strain, MKP103, requires LPS-core and Wzi for complete CPS retention.

WT and transposon mutants (*waaL::cat*, *wabG::cat*, *waaC::cat* and *wzi::cat*) of *K. pneumoniae* strain MKP103 were cultured in LB and their cell-associated CPS and cell-free EPS abundance and growth kinetics were analyzed. **(A)** Total CPS abundance was determined by measuring uronic acid content in total crude CPS extracts and normalized to OD_{600}. **(B)** Cell-associated CPS and cell-free EPS abundance were determined by uronic acid quantification. Cell-associated CPS was calculated by subtracting cell-free EPS from total CPS abundance. **(C)** The percentage of total CPS retained on the bacterial cell surface was calculated as the ratio of cell-associated CPS to total CPS shown in (B). Data presented are the mean, and error bars represent the standard error of the mean. Statistical significance was determined by comparing each mutant to WT using (A,C) one-way ANOVA with Dunnett's post-hoc test and (B) two-way ANOVA with Dunnett's post-hoc test. *P*-values are displayed above the bars; ** indicates $p \leq 0.01$, # indicates $p \leq 0.0001$. Experiments were performed three independent times, each in biological triplicate (n = 9).

bioRxiv preprint doi: https://doi.org/10.64898/2026.10.30.762281; this version posted October 31, 2026. The copyright holder for this preprint (which was not certified by peer review) is the author/funder, who has granted bioRxiv a license to display the preprint in perpetuity. It is made available under a CC-BY 4.0 International license.

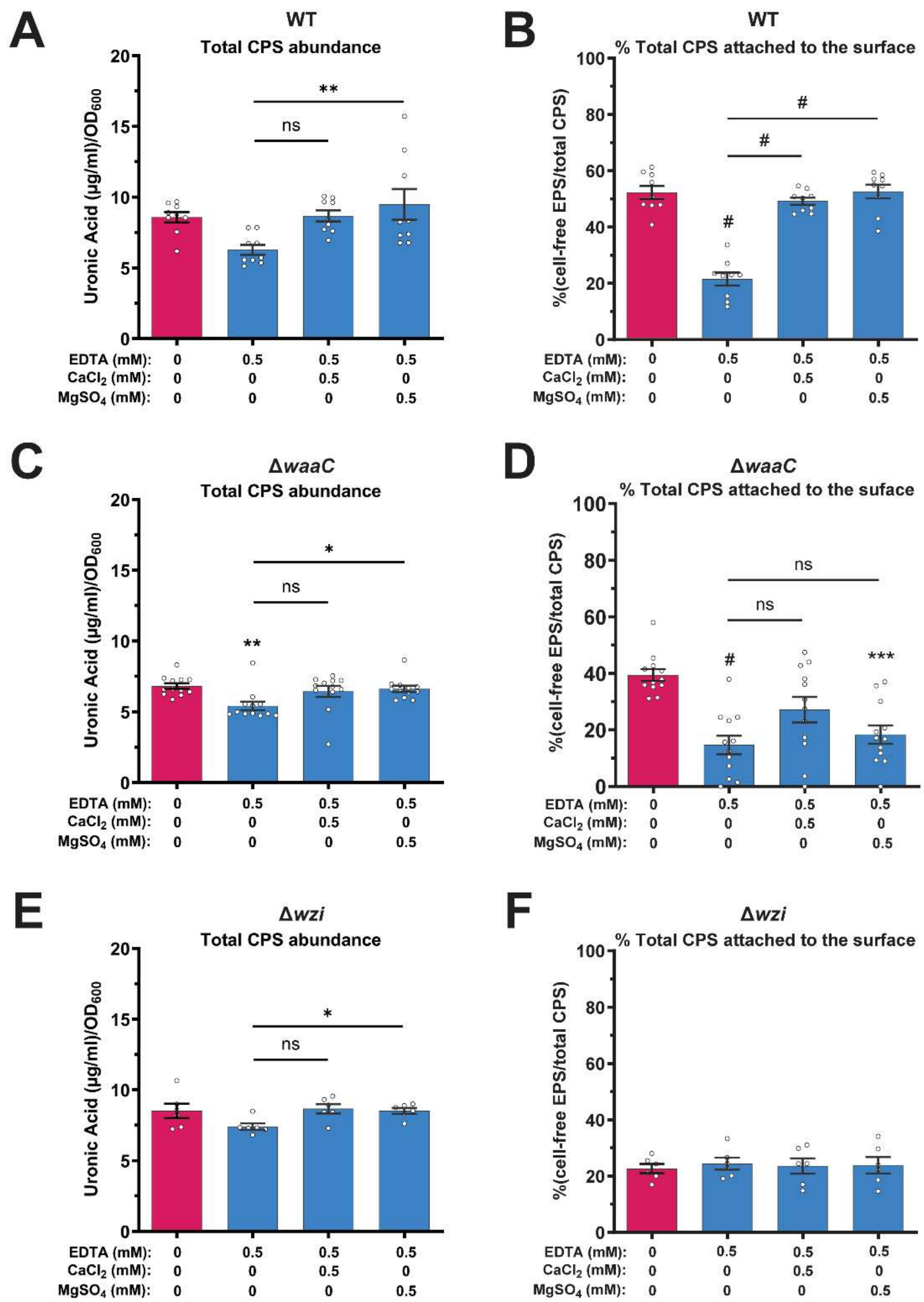

S4 Fig: Ca^{2+} and Mg^{2+} supplementation restores EDTA-induced loss of CPS retention in WT, but not in Δ*waaC* or Δ*wzi*.

KPPR1 **(A–B)** WT, **(C–D)** Δ*waaC* and **(E–F)** Δ*wzi* were cultured in LB treated with EDTA alone or EDTA supplemented with $CaCl_2$ or $MgSO_4$. **(A,C,E)** Total CPS abundance was determined by measuring uronic acid content in total crude CPS extracts and normalized to OD_{600}. **(B,D,F)** The percentage of total CPS retained on the bacterial cell surface was calculated as the ratio of cell-

bioRxiv preprint doi: https://doi.org/10.64898/2026.10.30.762281; this version posted October 31, 2026. The copyright holder for this preprint (which was not certified by peer review) is the author/funder, who has granted bioRxiv a license to display the preprint in perpetuity. It is made available under a CC-BY 4.0 International license.

associated CPS to total CPS shown in (A,C,E). Data presented are the mean, and error bars represent the standard error of the mean. Statistical significance was determined by two-way ANOVA with Šídák multiple-comparisons test, comparing (i) EDTA-only and EDTA+cation conditions to the untreated condition (*p*-values shown above each bar) and (ii) EDTA+cation conditions to the EDTA-only condition (*p*-values shown by connected lines); * $p \leq 0.05$, ** $p \leq 0.01$, *** $p \leq 0.001$, # $p \leq 0.0001$. Experiments were performed three independent times, each in biological triplicate ($n = 9$).

bioRxiv preprint doi: https://doi.org/10.64898/2026.10.30.762281; this version posted October 31, 2026. The copyright holder for this preprint (which was not certified by peer review) is the author/funder, who has granted bioRxiv a license to display the preprint in perpetuity. It is made available under a CC-BY 4.0 International license.

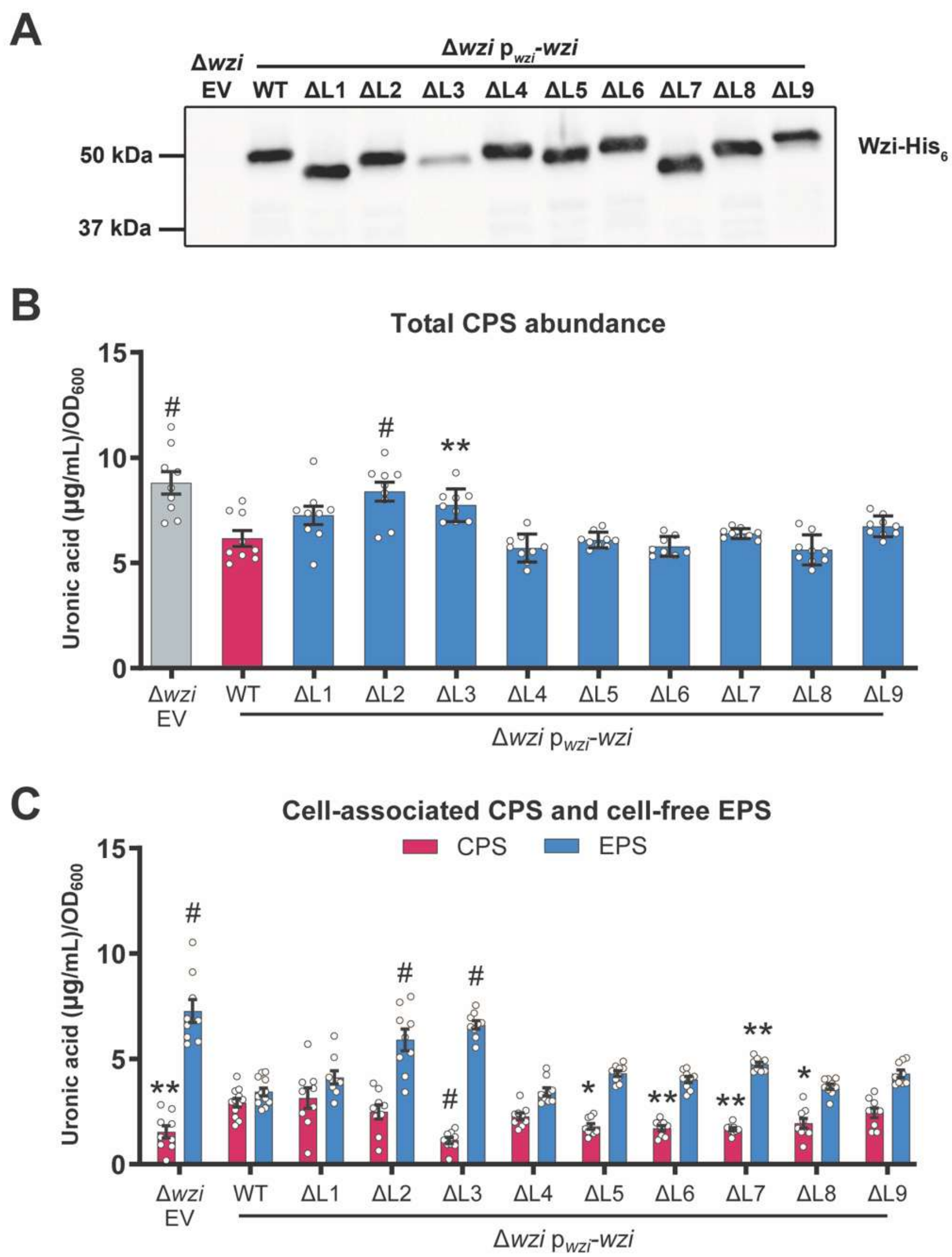

S5 Fig: Cell-associated CPS and cell-free EPS of various Wzi loop substitution variants.

KPPR1 Δ*wzi* with pBBR1-MCS5empty vector (EV) or the vector expressing WT or loop-substituted *wzi* (p_{wzi}-*wzi*) were incubated in LB. Wzi protein expression was examined and measured for total CPS abundance and cell-free EPS abundance. **(A)** Total cell lysate was prepared from an overnight culture of each strain, resolved by SDS-PAGE and subjected to Western blot, probing for his-tagged Wzi using mouse anti-his HRP-conjugated antibody. **(B)** Total CPS abundance was determined by measuring uronic acid content in total crude CPS extracts and normalized to OD_{600}. **(C)** Cell-free EPS abundance was determined by measuring uronic acid content is cell-free supernatant, whereas cell-associated CPS was calculated by subtracting cell-associated EPS from total CPS abundance presented in (A). The corresponding '% total CPS

bioRxiv preprint doi: https://doi.org/10.64898/2026.10.30.762281; this version posted October 31, 2026. The copyright holder for this preprint (which was not certified by peer review) is the author/funder, who has granted bioRxiv a license to display the preprint in perpetuity. It is made available under a CC-BY 4.0 International license.

attached to the cell surface' is presented in Fig 3F. Data presented are the mean, and error bars represent the standard error of the mean. Statistical significance was determined by comparing strains expressing loop-substituted *wzi* or EV to the strain expressing WT *wzi*, using (A) one-way ANOVA with Dunnett's post-hoc test or (C) two-way ANOVA with Dunnett's post-hoc test. *P*-values are displayed above each bar; * indicates $p \leq 0.05$, ** indicates $p \leq 0.01$, # indicates $p \leq 0.0001$. Experiments were performed three independent times, each with biological triplicate ($n = 9$).

bioRxiv preprint doi: https://doi.org/10.64898/2026.10.30.762281; this version posted October 31, 2026. The copyright holder for this preprint (which was not certified by peer review) is the author/funder, who has granted bioRxiv a license to display the preprint in perpetuity. It is made available under a CC-BY 4.0 International license.

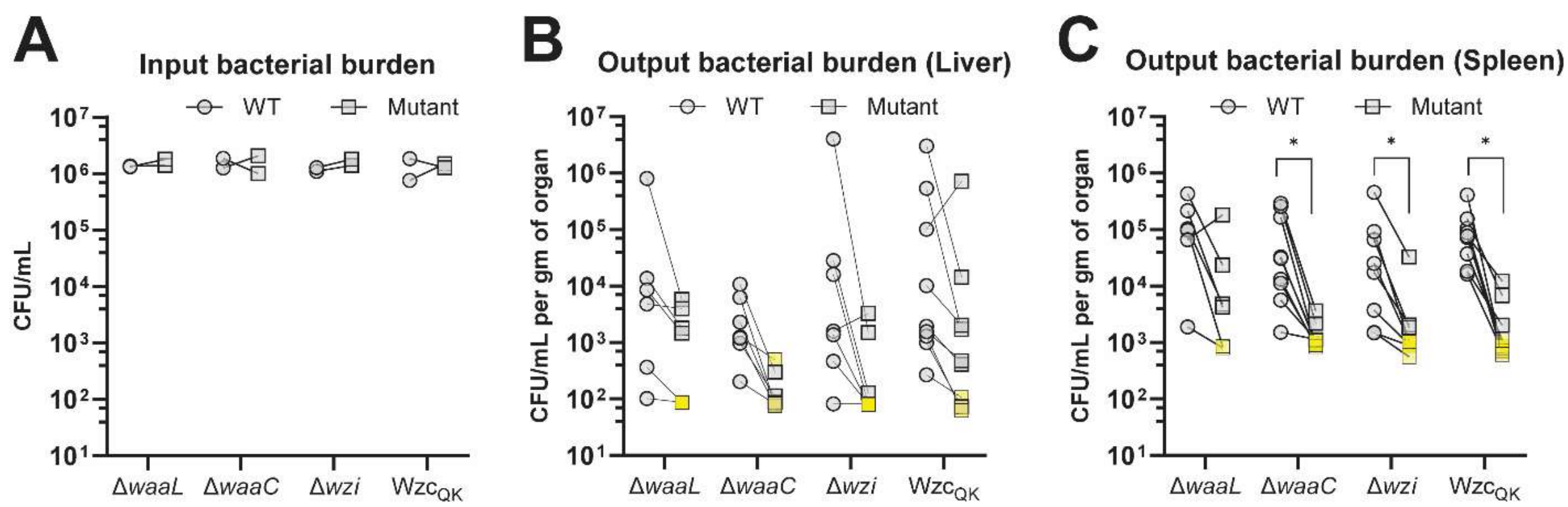

S6 Fig: *K. pneumoniae* CPS attachment mutants exhibit reduced liver and splenic burden following transient bacteremia.

(A) C57BL/6 mice were infected by tail vein injection with a mixture of KPPR1 WT and mutant at 1:1 ratio (10^5 CFU/mL each). **(B–C)** After 24 hours, bacterial burden in the liver and spleen were enumerated by differential plating. The data presented here are the absolute CFU/mL counts used to calculate $\log_{10}$ competitive index in Fig 5 using Equation 2. Each dot represents the CFUs of WT or a mutant. The paired input or output CFUs enumerated from the same mouse are connected by a line. CFUs below the detection limit are indicated by crossed symbol colored in yellow. **(B–C)** Statistical significance was determined using multiple Wilcoxon test with Holm-Šídák correction and calculated by comparing CFU of mutant to WT from the respective group. *P*-values are displayed above each comparison. * $p \leq 0.05$.
